# Updated guidelines for gene nomenclature in wheat

**DOI:** 10.1007/s00122-023-04253-w

**Published:** 2023-03-23

**Authors:** S. A. Boden, R. A. McIntosh, C. Uauy, S. G. Krattinger, J. Dubcovsky, W. J. Rogers, X. C. Xia, E. D. Badaeva, A. R. Bentley, G. Brown-Guedira, M. Caccamo, L. Cattivelli, P. Chhuneja, J. Cockram, B. Contreras-Moreira, S. Dreisigacker, D. Edwards, F. G. González, C. Guzmán, T. M. Ikeda, I. Karsai, S. Nasuda, C. Pozniak, R. Prins, T. Z. Sen, P. Silva, H. Simkova, Y. Zhang, the Wheat Initiative

**Affiliations:** 1 School of Agriculture, Food and Wine, Waite Research Institute, University of Adelaide, Glen Osmond, SA 5064 Australia; 2 School of Life and Environmental Sciences, University of Sydney, Plant Breeding Institute, 107 Cobbitty Road, Cobbitty, NSW 2570 Australia; 3 John Innes Centre, Norwich Research Park, Norwich, NR4 7UH UK; 4 Plant Science Program, Biological and Environmental Science and Engineering Division, King Abdullah University of Science and Technology, Thuwal, 23955-6900 Saudi Arabia; 5 Department of Plant Science, University of California, Davis, CA 95616 USA; 6 Departamento de Biología Aplicada, Facultad de Agronomía (CIISAS, CIC-BIOLAB AZUL, CONICET-INBIOTEC, CRESCA), Universidad Nacional del Centro de La Provincia de Buenos Aires, Av. República Italia 780, C.C. 47, (7300), Azul, Provincia de Buenos Aires Argentina; 7 Institute of Crop Science, National Wheat Improvement Centre, Chinese Academy of Agricultural Sciences, 12 Zhongguancun South St, Beijing, 100081 China; 8 N.I. Vavilov Institute of General Genetics, Russian Academy of Sciences, Moscow, Russia 119991; 9 International Maize and Wheat Improvement Center (CIMMYT), Apdo Postal 6-641, Mexico, D.F., Mexico; 10 USDA-ARS Plant Science Research, North Carolina State University, William Hall 4114A, Raleigh, NC 27695 USA; 11 NIAB, 93 Lawrence Weaver Road, Cambridge, CB3 0LE UK; 12 Council for Agricultural Research and Economics (CREA), Research Centre for Genomics and Bioinformatics, Via S. Protaso, 302, 29017 Fiorenzuola d’Arda, PC Italy; 13 School of Agricultural Biotechnology, Punjab Agricultural University, Ludhiana, 141 004 India; 14 Estación Experimental de Aula Dei (EEAD-CSIC), Zaragossa, Spain; 15 School of Biological Sciences, University of Western Australia, Perth, 6009 Australia; 16 Instituto Nacional de Tecnología Agropecuaria (INTA), EEA Pergamino, y Centro de Investigaciones y Transferencia del Noroeste de la Provincia de Buenos Aires (CITNOBA, CONICET-UNNOBA-UNSADA), Ruta 32. Km 4.5, CP 2700, Pergamino, Buenos Aires Argentina; 17 Department of Genetics, School of Agricultural and Forest Engineering, Universidad de Córdoba, Córdoba, Spain; 18 Agroecosystem and Crop Breeding Group, Western Region Agricultural Research Center, Fukuyama, Hiroshima 721-8514 Japan; 19 Centre for Agricultural Research, ELKH, 2462 Martonvasar, Hungary; 20 Laboratory of Plant Breeding, Graduate School of Agriculture, Kyoto University, Kyoto, 606-8224 Japan; 21 Crop Development Centre and Department of Plant Sciences, University of Saskatchewan, 51 Campus Drive, Saskatoon, SK S7N 5A8 Canada; 22 CenGen Pty Ltd., Worcester, 6850 South Africa; 23 Department of Genetics, Stellenbosch University, Matieland, 7602 South Africa; 24 Crop Improvement and Genetics Research Unit, USDA-ARS, 800 Buchanan St, Albany, CA 94710 USA; 25 Programa Nacional de Cultivos de Secano, Instituto Nacional de Investigación Agropecuaria (INIA), Estación Experimental La Estanzuela, 70006 Colonia, Uruguay; 26 Institute of Experimental Botany of the Czech Academy of Sciences, Šlechtitelů 31, 779 00 Olomouc, Czech Republic; 27 State Key Laboratory of Genetic Engineering, Collaborative Innovation Center of Genetics and Development, Institute of Plant Biology, School of Life Sciences, Fudan University, Shanghai, 200438 China; 28 The Wheat Initiative, 14195 Berlin, Germany

## Abstract

**Key message:**

Here, we provide an updated set of guidelines for naming genes in wheat that has been endorsed by the wheat research community.

**Abstract:**

The last decade has seen a proliferation in genomic resources for wheat, including reference- and pan-genome assemblies with gene annotations, which provide new opportunities to detect, characterise, and describe genes that influence traits of interest. The expansion of genetic information has supported growth of the wheat research community and catalysed strong interest in the genes that control agronomically important traits, such as yield, pathogen resistance, grain quality, and abiotic stress tolerance. To accommodate these developments, we present an updated set of guidelines for gene nomenclature in wheat. These guidelines can be used to describe loci identified based on morphological or phenotypic features or to name genes based on sequence information, such as similarity to genes characterised in other species or the biochemical properties of the encoded protein. The updated guidelines provide a flexible system that is not overly prescriptive but provides structure and a common framework for naming genes in wheat, which may be extended to related cereal species. We propose these guidelines be used henceforth by the wheat research community to facilitate integration of data from independent studies and allow broader and more efficient use of text and data mining approaches, which will ultimately help further accelerate wheat research and breeding.

**Supplementary Information:**

The online version contains supplementary material available at 10.1007/s00122-023-04253-w.

## Introduction

Wheat, the world’s most widely grown cereal, produces grain that accounts for ~ 20% of the protein and calories consumed globally (www.fao.org; Shiferaw et al. 2013). The sustainability of wheat yields relies on breeding, which strives to maintain and enhance grain production by improving traits such as pathogen resistance, tolerance to abiotic stresses, end-use quality, and yield potential, among others. Breeders achieve these productivity gains by identifying genetic variation for these agronomically important traits, with beneficial alleles being selected, and sometimes fixed, among elite cultivars. Similarly, natural and human selection for this genetic variation contributed to the domestication of wheat from its wild relatives. Examples of such variation includes the *Q* allele of domesticated wheat that confers a subcompact spike with free-threshing grain, *Reduced height* alleles, that decrease height and lodging by suppressing sensitivity to phytohormones, and the photoperiod insensitive alleles of *PHOTOPERIOD-1* that promote flowering under shorter daylengths (Beales et al. 2007; Debernardi et al. 2017; Peng et al. 1999; Simons et al. 2006).

The need to learn more about the genetic basis of domestication and subsequent breeding has encouraged analyses of the wheat genome. Historically, this work has included investigation of wheat’s genetic ancestry and hybridisation of the sub-genome species, the discovery of synteny among cereal genomes, and the development of aneuploid lines to identify chromosomal segments containing genes responsible for key traits (Kihara 1944; Sears 1948, 1954; Gale and Devos 1998; Moore et al. 1995; Tsunewaki 2015). Traditionally, gene designations in wheat were based on morphological or phenotypic features that were genetically mapped as discrete genetic units or loci. Hence, a locus is defined as a chromosomal site of variable size at, or within which, is located a gene, a restriction site, a breakpoint, an insertion, or other distinguishable feature (Woodhouse et al. 2021). More recently, the assembly of reference and pangenome sequences for wheat, its progenitors and closely related species, and an improved understanding of gene expression has facilitated identification of molecular sequences underlying a locus (Gaurav et al. 2022; International Wheat Genome Sequencing et al. 2018; Ling et al. 2018; Ramirez-Gonzalez et al. 2018; Walkowiak et al. 2020). These advances have provided an exciting step-change in wheat science and have encouraged research to analyse gene function in wheat based on homologues described in other species. Together with the generation of mutant populations and improved transformation capabilities that have enhanced our ability to examine gene function (Debernardi et al. 2020; Ishida et al. 2015; Krasileva et al. 2017), we anticipate there will be a vast number of new loci discovered and reported in coming years. Based on this anticipated expansion of reported loci, we propose an updated set of guidelines for designating names and symbols of genes for adoption by the wheat community.

This work represents a contribution to set guidelines for gene nomenclature across the Triticeae (e.g. *Triticum aestivum*, *Triticum turgidum*, *Triticum uratu*, *Aegilops tauschii, Aegilops speltoides, Triticum timopheevii* and beyond). These guidelines follow those established for the Wheat Gene Catalogue (McIntosh et al. 2013), an initiative established and supported by the now disbanded International Wheat Genetics Symposium from 1968 to 2017. The guidelines have been updated here to provide examples to accommodate recent advances in our understanding of wheat genomes. This organisation is consistent with previous wheat nomenclature (McIntosh et al. 2013) and that of rice (McCouch et al. 2008). It would indeed be desirable to have a common language to designate the genes in wheat, barley, oat, and rye, given the shared breeding targets for these cereals. The close phylogenetic relationships among the Triticeae easily allows identification of orthologues (i.e. a homologous gene that evolved from a common ancestral gene by speciation) and a common gene nomenclature criterion could help define gene families for investigation at the sequence and functional level.

**Fig. 1:**
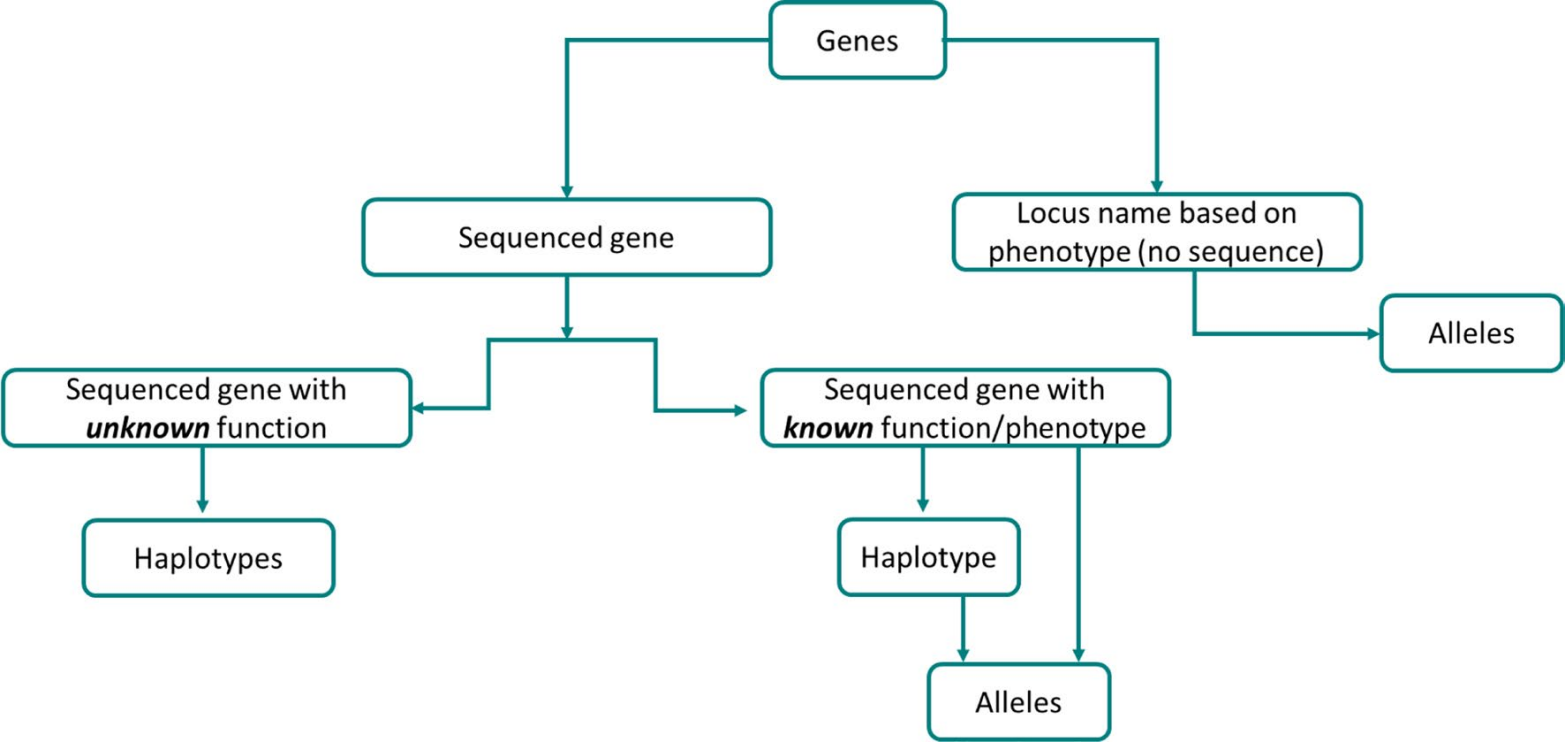
General process for navigating wheat gene nomenclature; based on McCouch et al. (2008). Whereas a gene may be defined as a segment of DNA with a known or predicted function or phenotype and alleles may be defined as variations in a gene sequence, we have adopted the precedence of McCouch et al. (2008) in distinguishing “alleles” based on function/phenotype from sequence variants or “haplotypes”

Here, we describe the updated guidelines for the use of gene symbols in wheat based on (i) locus designations identified based on morphological or phenotypic features (In “Recommended rules for symbolisation of genes conferring morphological, physiological and grain quality traits, proteins, and disease/pest resistance” section) or (ii) gene nomenclature once the underlying gene has been identified (i.e. cloned) and for genes identified by sequence similarity with other species (often without a reported phenotype) (In "Guidelines for nomenclature of biochemical molecular loci in wheat and related species" section) (Fig. 1). We also provide guidelines for the naming of related genetic entities and macromolecules, including gene complexes (In "Gene complexes" section), pseudogenes (In "Pseudogenes" section), proteins (In "Proteins" section), DNA markers (In "Symbols for DNA markers" section), quantitative trait loci (in "Symbols for loci and alleles controlling quantitative characters" section), genes for reaction to pests and pathogens (In "Guidelines for nomenclature of genes for reaction to pathogenic diseases and pests" section), and grain proteins and enzymes (In "Guidelines for nomenclature of genes underlying variation in proteins and enzymes" section). The Wheat Initiative (https://www.wheatinitiative.org/), which is supported by The Group of Twenty (G20), endorses these recommendations and strongly encourages the wheat science community to adopt the updated guidelines.

## Recommended rules for symbolisation of genes conferring morphological, physiological and grain quality traits, proteins, and disease/pest resistance

### Gene name

The name should briefly describe the principal characteristics associated with the phenotype rendered due to mutant or allelic forms of the locus, e.g. genes involved in the vernalisation response of flowering have been named *VERNALISATION* (*VRN*) and those that reduce plant height have been named *REDUCED HEIGHT* (*RHT*). Apart from a few classic gene names (e.g. *Q*, *C*, *s*), the use of single letter designations should be avoided.

### Formatting of gene names

Roman script and Arabic numbers should be given preference in naming hereditary factors. Symbols of hereditary factors, derived from their original names, should be written in italics, or in Roman letters of distinctive type.

Locus names should be written in uppercase italics (e.g. *SR9, VRN-A1)*; the name and symbol of a dominant or semi-dominant allele should begin with an uppercase first letter (*Sr9a*) and those of a recessive allele with a lowercase letter (*sr9* or *sr9a*) (for exceptions, see also special rules for symbolising biochemical and DNA loci in “Biochemical nomenclature” and “Genes specifying the structure of similar macromolecules” sections, and host:pathogen/pest systems in "Guidelines for nomenclature of genes for reaction to pathogenic diseases and pests" section). Similarly, for the *VRN-A1* locus, the dominant or semi-dominant alleles should be *Vrn-A1* and the recessive alleles should be *vrn-A1*.

So far as possible all letters and numbers used in symbolisation should be written on one line; superscripts or subscripts should be avoided, except when used to designate wheat wild relative genomes (e.g. the *EARLINESS *PER SE locus from *Triticum monococcum (A*^*m*^ genome) is designated as *EPS-A*^*m*^*1*).

### Symbolisation

The plus sign (+) will not be used in symbolisation of hereditary factors in wheat. In instances where a heterozygous condition is being described for a single gene, or where alleles are codominant, then the genotype should be written with each allele separated by a slash. For example, a heterozygous genotype at *VRN-A1* should be presented as *Vrn-A1/vrn-A1*; a heterozygous codominant genotype can be presented as *Sr13a/Sr13b*.

### Genes with similar phenotypic effects

Two or more genes having phenotypically similar effects should be designated by a common basic symbol. Non-allelic loci (mimics, polymeric genes, etc.) will be designated in accordance with two procedures:

i. In sequential polymeric gene series where an Arabic numeral immediately follows the gene symbol, e.g. *SR9, SR10, SR11*.
ii. In orthologous sets where the basic symbol is followed by a hyphen (“-”) followed by the locus designation taking the form of the accepted genome symbol and a homoeologous set number represented by an Arabic numeral, e.g. *VRN-A1* designates the A-genome member of the first vernalisation (*VRN*) set. *VRN-B1* would designate the B-genome member of this first *VRN* set. On the other hand, *VRN-A2* designates the A-genome member of the second *VRN* set. The order of these numbers should refer to their sequence of discovery, and if more than one set is reported in a single publication, they should follow an ascending order based on chromosome location. Importantly, chromosome names should not be included in the basic symbol, e.g. *VRN-A1*, which is located on chromosome 5A should not be named *VRN-5A*, *VRN1-5A*, or similar. Different alleles, or alleles of independent mutational origin, are designated by a lowercase Roman letter following the locus name or number, e.g. *Sr9a, Vrn-A1a* (see in “Alleles” section).

#### Temporary symbol designations

Where linkage data are not available or are inconclusive, provision has been made for temporary symbols that can be used to name loci prior to more comprehensive genetic analyses or where appropriate germplasm is not freely available. These names shall consist of the basic locus symbol (*SR*) followed by an abbreviation (maximum of 3 letters) for the line or stock (e.g. *Fr* for Federation) and an Arabic number referring to the locus, e.g. *SRFr1, SRFr2*, etc., refer to two loci for reaction to the fungus *Puccinia graminis* in the wheat cultivar Federation (Fr). It is recommended that records of other laboratories (e.g. *SRFr3*) be checked against earlier numbers either phenotypically or genetically.

### Inhibitors, suppressors, and enhancers

Inhibitors, suppressors, and enhancers can be prefixed by the symbols *I*, *Su* and *En*, or by *i*, *su* and *en* if they are recessive, followed by the symbol of the allele affected. For example, the *Pairing homoeologous 1* (*Ph1*) locus is suppressed by genes on chromosomes 3S and 7S of *Aegilops speltoides*, and the suppressing alleles were named *Su1-Ph1* and *Su2-Ph1*, respectively (Dvorak et al. 2006).

### Linkage groups and syntenic regions of the genome

In wheat and related species, linkage groups and corresponding chromosomes are designated by an Arabic numeral (1–7) followed by genome designated by an uppercase Roman letter, i.e. for hexaploid wheat of species *aestivum* which contains A, B and D sub-genomes (Morris and Sears 1967; Kihara 1944; Tsunewaki 2015), 1A-7D. This system supersedes the original designations using Roman numerals, i.e. I–XXI. Chinese Spring was accepted as having the standard chromosome arrangement. Chromosome arms (or telocentric chromosome derivatives) are designated S (short) or L (long), based on the relative arm length within the chromosome. In the case of equal arm length, they are arbitrarily designated S or L based on homoeology with the short or long arms of other chromosomes of their homoeologous group (see Workshop I, Proceedings of the 7th International Wheat Genetics Symposium), e.g. the arm designations for chromosome 7D were reversed based on homoeology (Werner et al. 1992). When wheat chromosomes are represented vertically in diagrams, the convention is to place the short arm on top and the long arm beneath.

### Genetic formulae

Genetic formulae may be written as fractions, with the maternal alleles given first or above. Each fraction corresponds to a single linkage group. For example, *Aa* or *A/a* for a heterozygote derived from cross *AA* × *aa* with the genotype of the female parent is written first. Similarly, a heterozygous plant resulting from the cross between *Vrn-A1* and *vrn-A1* can be written as *Vrn-A1/vrn-A1*. Linkage of loci can be indicated by underlining, i.e. *AB/ab* when the dominant alleles are in coupling, or *Ab/aB* when in repulsion.

### Chromosomal aberrations

Chromosomal aberrations should be indicated by the abbreviations Df for deficiency, Dp for duplication, Inv for inversion, T for translocation and Tp for transposition. In wheat, there are numerous genes derived from related species by introgression. Once present in a chromosome capable of pairing with a wheat chromosome, those genes will be designated as wheat genes, e.g. wheat gene *SR26* is present in a chromosome 6A-6Ag translocation involving a region of chromosome 6Ag from *Thinopyrum ponticum*. If the identity of that gene in the donor species (e.g. *Thinopyrum ponticum*) is known, its name should be treated as a synonym. Such genes in different instances may reside at different locations; one location may be taken as standard and other locations can be considered transpositions relative to the designated standard, e.g. when a gene does not reside in its standard chromosome position, the new chromosome designation may be given in brackets following the gene designation; *HP(Tp6D)* could refer to a line carrying the introgressed “hairy neck” (hairy peduncle) gene on chromosome 6D (Sears 1967) instead of 4B, which is taken as standard (Driscoll and Sears 1965). Alternatively, the chromosome involved may be described as a translocation. Guidelines for the description of translocated chromosomes both within wheat, and between wheat and alien chromosomes, are provided in Koebner and Miller (1986).

### Genome formula

The zygotic chromosome number is indicated by 2*n*, the gametic number by *n* and the basic number by *x*, e.g. 2*n* = 6*x* = 42 for bread wheat.

### Extra-chromosomal units

Symbols for genes in extra-chromosomal units (mitochondria, plastids, chloroplasts) should be prefixed with a characteristic, underlined, defining symbol such as *Mt*, *Pt*, or *Cp* preceding the gene name.

## Guidelines for nomenclature of biochemical molecular loci in wheat and related species

### Biochemical nomenclature

Biochemical nomenclature should be in accordance with the rules of the Joint Commission of Biochemical Nomenclature (JCBN) of the International Union of Pure and Applied Chemistry. The nomenclature recommended by the JCBN is published periodically in major international biochemical journals, such as the Journal of Biological Chemistry and the European Journal of Biochemistry. Also, for enzymes, the publication Enzyme Nomenclature (Anonymous 1979, 1986) may be consulted. Enzymes and other macromolecules have both formal and trivial names. The formal name should be given the first time a macromolecule is mentioned in a publication; the trivial name or an abbreviated name may be used subsequently. For example, ADH is the commonly used abbreviation for aliphatic alcohol dehydrogenase (formally, E.C.1.1.1.1; Alcohol: NAD + oxireductase).

### Basic symbol

#### Gene nomenclature for a cloned gene

**Fig. 2:**
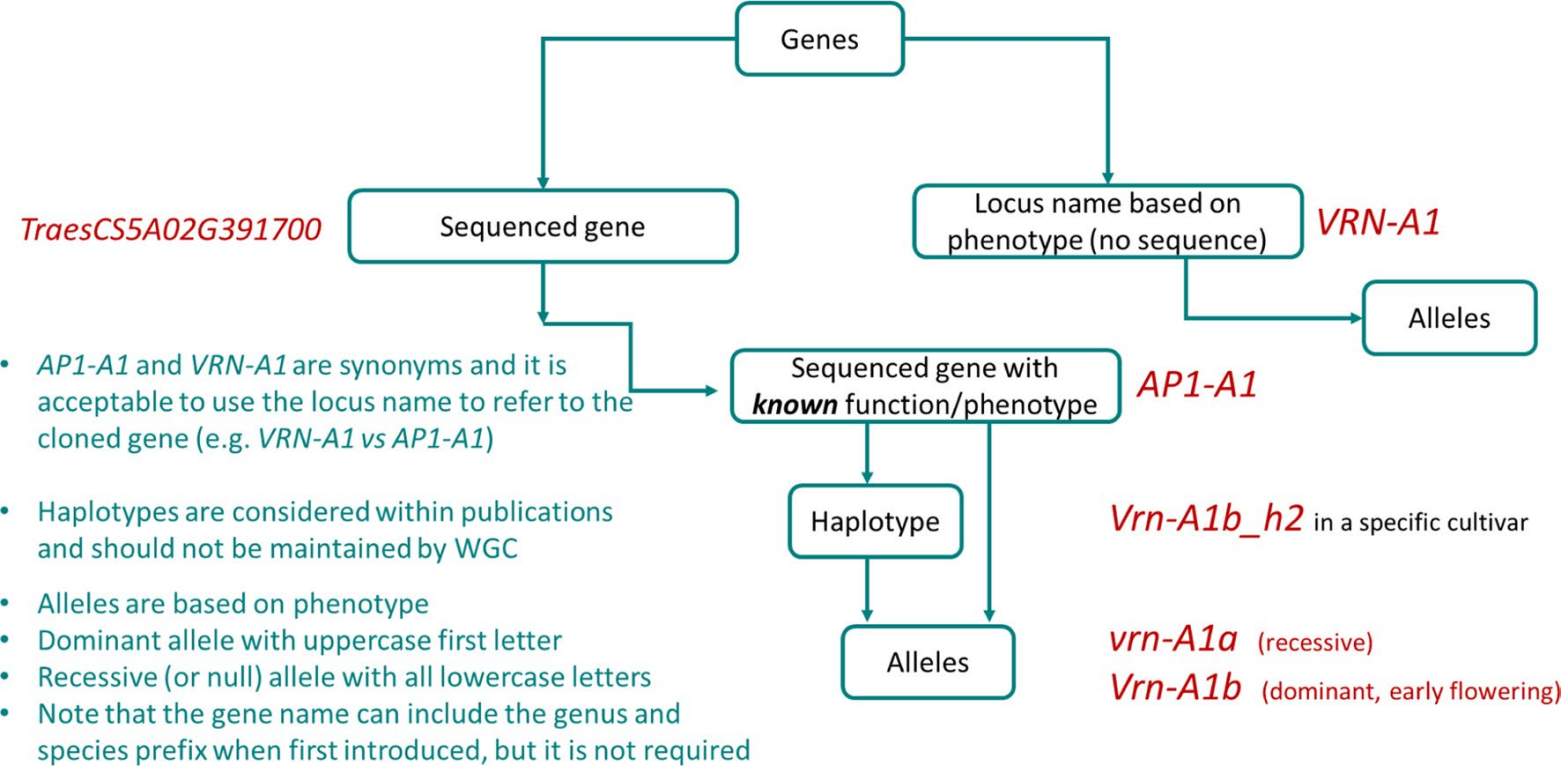
Example of a gene with known phenotype (*VRN-A1*) and later cloned (*TaAP-A1*). WGC: Wheat Gene Catalogue

The basic symbol for a gene should consist of a two-, three- or four-letter abbreviation of the trivial name of the enzyme, protein, or other macromolecule affected. The name should briefly describe the principal characteristics associated with a biochemical function of the gene product. All letters for a gene should be uppercase and italicised and alleles should have uppercase first letters if dominant, or all lowercase if recessive. For example, the wheat *VRN-A1* locus on the A genome is encoded by the wheat *APETALA1-like* gene (*AP1-A1*) (Yan et al. 2003). It is acceptable to use the locus name to refer to the cloned gene (e.g. the *VRN-A1* locus is encoded by the gene *AP1-A1*, but the locus name *VRN-A1* is more commonly used). Proteins can be symbolised in uppercase non-italicised style corresponding to the allele name (SR9a, AP1-A1a). The gene name can include the genus and species prefix when first introduced or when required to distinguish the same (orthologous) gene from multiple species (*TaAP1-A1* from *T. aestivum* and *TtAP1-A1* from *T. turgidum*), but is not required for continued references to the same gene. The associated gene model identifier (e.g. *TraesCS5A02G391700* for *AP1-A1* in the wheat reference genome of cultivar Chinese Spring) should be included (when available) in publications or by advice (with source of information) to a catalogue curator. Both gene model and transcript isoform (e.g. *TraesCS5A02G391700.1*) should be written in italics. Sequence variants within genes already named based on the plant/molecular phenotype or named by sequence variation will be called alleles or haplotypes, respectively (see in “Haplotypes” and “Alleles” sections). An example is shown in Fig. 2 and Table 1.

**Table 1:** Example of gene nomenclature for the *VRN-A1* locus

Name	Description
*VRN-A1*	Designation for the first locus affecting *VERNALISATION* response in wheat, located on the A genome (all uppercase and italics)
*TaAP1-A1*	Name of the gene underlying the *VRN-A1* locus (RefSeqv1.1 *TraesCS5A02G391700*). *TaAP1-A1* is the first (*1*) wheat homologue, located on the A genome, of the *Arabidopsis APETALA1-like* (*AP1*) gene (all uppercase and italics)^1^
*ap1-A1a* *vrn-A1a*	Name of the wild-type Chinese Spring allele (a recessive allele, so lowercase first letter). Note that the allele name (*vrn-A1*) can also be used, and is commonly used for this gene
*Vrn-A1b*	Name of allele which affects flowering time with respect to the wild-type Chinese Spring allele (e.g. *cv*. “Marquis” early allele with promoter and 5′ UTR variation). The allele is a dominant allele, so an uppercase first letter is used
*Vrn-A1b_h2*	Name of a haplotype with sequence variants present in the Marquis *Vrn-A1b* allele (Chinese Spring is *_h1*) and which has a vernalisation insensitive early flowering phenotype
VRN-A1 AP1*-*A1	Name of the protein encoded by the *VRN-A1* gene. Note that again the AP1-A1 nomenclature can be used for this protein (all uppercase, non-italicised)

^1^Note that the gene name can include the genus and species prefix when first introduced (e.g. *TaAP1-A1*), but it is not required

#### Genes identified based on homology to other plant species

**Fig. 3:**
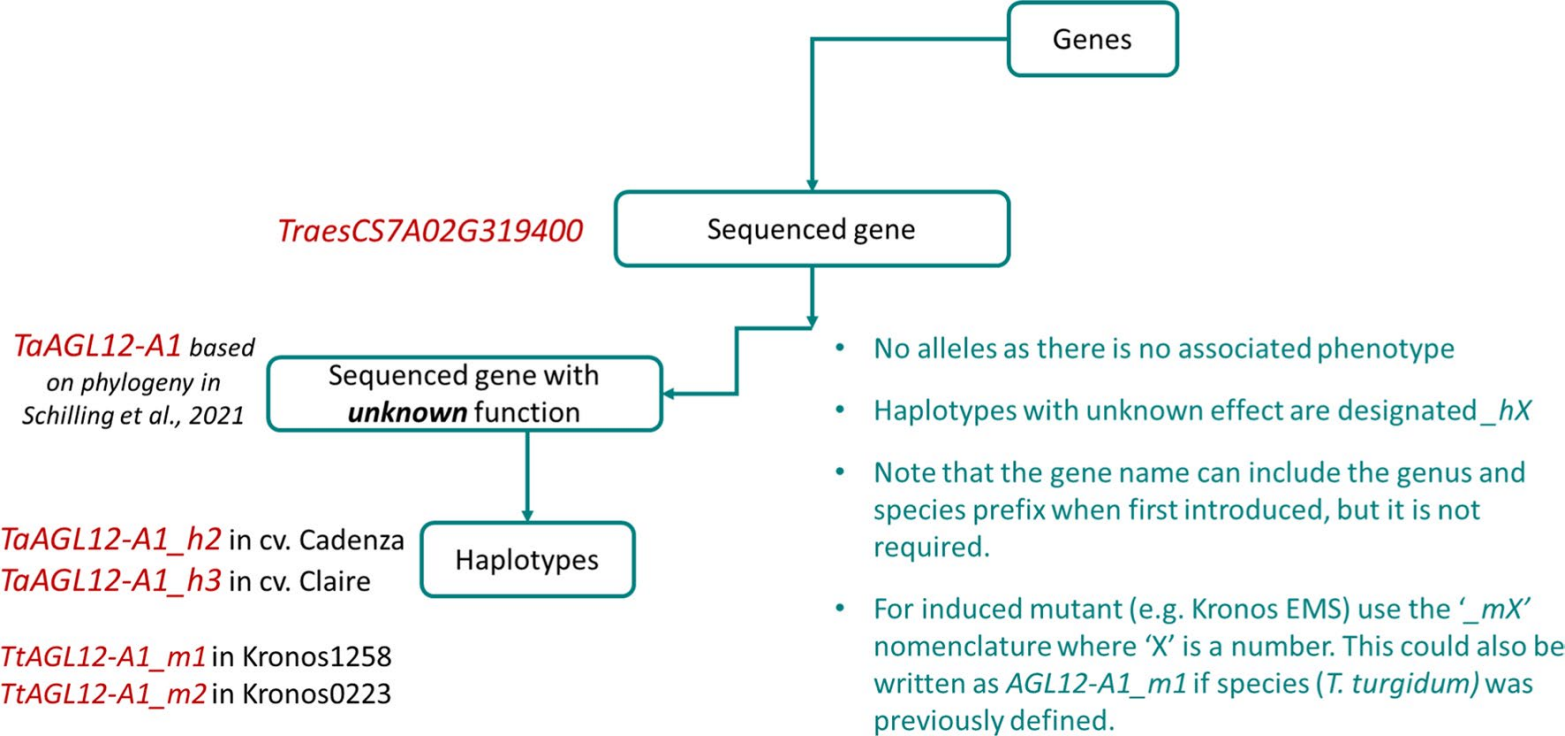
Example of a gene named based on sequence homology to a gene from another plant species. Here, the gene model *TraesCS7A02G319400* is named as *TaAGL12-A1*, based on phylogenetic analyses. Sequence variants are identified in two cultivars and in two ethyl methanesulfonate (EMS) mutants from cv. Kronos; these are referred to as haplotypes as they have no phenotype associated with them

Genes identified by homology (based on shared sequence similarity/phylogenetic analysis), and with no known associated phenotype in wheat, should adopt the gene symbol from the original species (Fig. 3). A two-letter prefix with the wheat species name can be used to distinguish it from the previously described homologous gene from another species, but is not part of the formal gene name; the name can include the prefix when first introduced but is not required for successive references to the gene. Likewise, we recommend, when possible, retention of the same gene number as designated in the original species, e.g. the common wheat (*Triticum aestivum*) homologues of the *Arabidopsis thaliana SEPALLATA1* (*SEP1*) gene should be designated as *SEP1-1*, *SEP1-2*, etc., with the A, B and D genome homeologues designated as *SEP1-A1, SEP1-B1* and *SEP1-D1* (e.g. Schilling et al. 2020)*.* When the gene name used ends in a number (e.g. *SEP1*) and there are multiple paralogues of the gene in wheat, then the paralogue number should be separated from the gene name by a dash, e.g. *SEP1-1* and *SEP1-2* are wheat homologues of *SEP1* from *Arabidopsis*. In publications referring to the same gene from multiple species, the gene can be referred to with its genus and species prefix, e.g. *TaSEP1-1* for the *SEP1-1* homologue from *T. aestivum* and *TtSEP1-1* of *T. turgidum*. This homology-based assignment should be based on comprehensive molecular phylogenies which shall include, as a minimum, all related wheat and rice genes for the relevant sequence.

#### Priority of names

When using another species name, which name should be used if there are different names in different species? A gene sequence might have multiple designations, for example, the rice *MADS22* gene is equivalent to the barley *BM10* gene and the maize *ZMM19* gene (which underlies the *TUNICATE1* locus). In addition, this gene sequence has previously been named as *SVP1* in wheat. We recommend that when possible, a single gene name takes priority and that alternative nomenclatures be mentioned within manuscripts to aid in cross-species comparisons. Where possible, historic published wheat names that have been assigned function should have priority. Alternatively, the gene name proposed should be based on the gene name whose functional characterisation is the closest to that being studied in wheat.

Authors referring to specific wheat genes in publications must cite the full gene name and symbol, as well as the gene model identifier (and transcript where relevant) from one of the genome annotations (e.g. Chinese Spring RefSeqv1.1 *TraesCSXX02GXXXXXX* gene models for hexaploid wheat and Svevo v1.0 *TRITDXXvXXXXXX* gene models for tetraploid wheat; Maccaferri et al. 2019). Where possible, the gene model identifier should be from a wheat line that contains an annotated version of the gene, where the transcript has been determined based on RNA-seq reads or complementary DNA sequence. As the high-quality reference genome assembly for wheat is from the cultivar Chinese Spring, its gene model should be used if it has the functional allele. Alternatively, if a gene is not annotated in Chinese Spring, or if it carries a non-functional allele, then a gene model identifier from another cultivar with a functional allele should be used where possible (e.g. a cultivar from the 10 + Wheat Genome Project; Walkowiak et al. 2020), along with the relevant genome assembly accession (e.g. GCA_900519105.1). If an altogether novel sequence is identified, this sequence should be annotated and deposited in one of the International Sequence Database Collaboration repositories (see www.insdc.org) with the accession number provided in the relevant publication.

### Genes specifying the structure of similar macromolecules

Non-allelic gene loci that specify the structure of similar non-enzymatic proteins, enzymes that catalyse the same or similar reactions, or of similar RNA molecules should be assigned the same basic symbol. The remainder of the symbol for each such gene should be formulated in accordance with one or other of two procedures, depending on whether evidence is available to assign the gene to a homologous set. Where designations extend beyond defined *Triticeae* genomes, the designation can be prefixed by a species abbreviation, e.g. *OsNAM1* is suggested as the rice orthologue of the wheat gene *NAM-A1*. Abbreviations for other species (e.g. *Ta* or *Tt* for *T. aestivum* or *T. turgidum,* respectively) can also be used when relevant for comparative genomics.

#### Genes that are members of a homoeologous set

The basic symbol should be followed by a hyphen (−), the accepted symbol for the genome to which the locus belongs and an homoeologous set number in the form of an Arabic numeral. The Arabic numeral indicates the order in which a particular gene or gene family member was identified and should not be confused with the chromosome on which it is found. For example, *FT-A1*, *FT-B1*, and *FT-D1* designate the A-, B-, and D-, genome members, respectively, of the first-designated homoeologous set of wheat homologues of the *Arabidopsis FLOWERING LOCUS T* (*FT*) gene. In the case of a single member set, the default number is 1.

Evidence regarding phylogenetic relationships among structural genes may be obtained by comparative studies of: (1) nucleotide sequences and other molecular properties of genes, (2) peptide sequences, (3) physical and/or biochemical properties of gene products, and (4) intra-chromosomal map positions and/or physical locations of genes in homoeologous chromosomes or segments. For an example of criteria, see Hart (1987). The evidence used to designate genes as members of a homoeologous set should be stated in the publication in which symbols for the locus are proposed.

#### Other loci

In the absence of evidence to assign a locus to an homoeologous set, that locus should be designated in a sequential series by an Arabic numeral. If evidence to assign the locus to an homoeologous set is obtained subsequently, the locus should be redesignated in accordance with the procedures in "Genes that are members of a homoeologous set" section. The same applies for genes identified by homology, e.g. if two *Arabidopsis SEP1* homologues are identified in wheat, their A genome copies would be *SEP1-A1* and *SEP1-A2*.

### Haplotypes

Haplotypes refer to DNA sequences of unspecified length and may include variable upstream and downstream regions, and these limits should be defined when reported. Haplotypes will take the form *_hX* following the relevant locus or allele designation; lowercase italicised *h* meaning haplotype, and *X* being a sequential numeral. Haplotypes represent sequence variants whose specific function is either (1) unknown or (2) is associated with a specific phenotype and hence can be related to an allele (Figs. 1, 2, 3). Haplotypes will be considered unique to the publications or projects from which they are reported, and lists will not be maintained as part of the Catalogue.

#### Haplotypes with unknown phenotypes

Such haplotypes should carry the uppercase italicised locus name followed by *_hX*. For example, a study investigates the allelic variation in the A genome wheat homologue of the Arabidopsis *BRASSINOSTEROID-INSENSITIVE 1* gene (*TaBRI-A1*). Four different sequences (haplotypes) are identified by investigating ± 2 kb up/downstream of *TaBRI-A1* in a panel of accessions. These four haplotypes, with unknown phenotypic effects, should be listed as *TaBRI-A1_h1* to *TaBRI-A1_h4* (Fig. 3).

#### Haplotypes associated with a specific phenotype

If a haplotype is identified and can be assigned a novel phenotype for a locus, then this haplotype will adopt an allele name (see description in “Alleles” section). If additional sequence variants are identified within a designated allele, these should carry the relevant allele name followed by *_hX*, e.g. *Sr9a_h1* and *sr9a_h1* describe the first DNA variants within the alleles that confer resistance and susceptibility, respectively. *Rht-A1a_h3* designates the third haplotype within the *Rht-A1a* allele (see further examples in “Alleles” section).

### Alleles

Alleles are based on phenotype. Phenotypes can either be “plant-based phenotypes”, in which case they are defined as changes in appearance, performance or responsiveness of the plant, or “molecular phenotypes” which are alterations in the biochemical, molecular function or physical properties, of a macromolecule, which is unique from the described characteristics of a reference macromolecule. A “molecular phenotype” could include sequence variants which lead to amino acid changes that impact on protein function, protein mass, or electrophoretic mobility (e.g. glutenin), such as those that are especially important for a grain quality parameter or a particular protein (see in "Guidelines for nomenclature of genes underlying variation in proteins and enzymes" section). It is important to note that synonymous mutations or polymorphisms which are used solely to discriminate sequences with genetic markers (e.g. KASP assay) are not considered alleles and should be designated as haplotypes. The exception to this guideline would be synonymous mutations that alter the expression of a gene, such as a microRNA complementary site (e.g. changes that occur in *Q*). Similarly, a locus containing a mutation that generates a phenotype by disrupting a *cis-*regulatory region (e.g. promoter, intronic region) can be considered an allele if the change affects gene expression. In publications, authors should make clear the basis of the plant or molecular phenotype that is being used to assign an allele.

Different natural existing alleles of a gene are designated by lowercase italic letters following the gene designation. For example, *Rht-B1a* and *Rht-B1b* are two alleles of the B genome copy of *RHT-1*. One accession should be designated the prototype genotype for each allele discovered, since variation that has not been detected by the methods used may be present within each allelic class. Currently, Chinese Spring is preferred as the prototype for allele “*a*”. If an allele in another genotype is found to be different from that in the prototype genotype and is shown to underlie a morphological/phenotypic difference with respect to the prototype “*a*” allele, it should be assigned a new lowercase italic letter and a prototype genotype designated. When referring to alleles, dominant, semi-dominant or co-dominant alleles should have the locus name with the first letter in uppercase (e.g. *Rht-B1b*), whereas recessive alleles, including null alleles, should have the locus name in all lowercase letters (*rht-B1a*). New alleles should be used in sequential alphabetical order. In situations of multiple allelism, the relevant alleles with an uppercase first letter can be used, and lowercase can be used for the null allele, where relevant.

Given that alleles are based on phenotype, novel alleles should only be assigned if they can be distinguished from known phenotypes (i.e. alleles) based on a specific phenotypic assay. The basis of this phenotype should be explained by authors when the allele is first described or published (e.g. plant-based, or molecular phenotypes as outlined above). Alternatively, if a sequence variant is identified for a known allele, but results in the same phenotype, then this will be designated as a haplotype within a specific allele. For example, the red glume (*RG*) locus has two historically defined alleles (*Rg1a* = red and *rg1b* = white). The cloning of *RG* identified several haplotypes of the underlying gene (each with a few amino acid substitutions) which all result in a red glume phenotype. Hence these haplotypes are all within the designated *Rg1a* allele and should be named *Rg1a_h1*, *Rg1a_h2*, etc. Likewise, multiple sequence variants (haplotypes) were identified which result in white glumes; these haplotypes should be named *rg1b_h1*, *rg1b_h2*, etc. (Abrouk et al. 2021).

### Induced mutants and gene-edited lines

As in the case of haplotypes (In “Genes with similar phenotypic effects” section), induced mutant versions (e.g. EMS mutagenesis or gene edited) of a particular locus will take the form *_mX* following the relevant gene or allele designation; lowercase italicised *m* meaning mutant, and *X* being a sequential numeral (Fig. 3, e.g. *AGL12-A1_m1*, *AGL12-A1_m2*). This nomenclature should be used for the independent mutant or gene-edited versions of a locus that are used to confirm gene function in relation to a particular trait; additional alleles generated by independent studies should be numbered in a consecutive ascending order. This system will facilitate the introduction of multiple mutant or gene edited lines for publication, which will not be curated in the wheat gene catalogue. Instead, we recommend that publications introducing such mutant or gene edited lines should include a table listing the multiple mutations and alleles.

When an induced mutant has been selected from a Targeting Induced Local Lesions in Genomes (TILLING) population, the mutant line should be referred to according to its identity within the population, e.g. Cadenza1715, Kronos2267. In cases where a TILLING line and its derivatives are subsequently investigated for a phenotype of interest, the underlying genes can be identified as described in “Basic symbol” section and the mutant line named according to the guidelines at the beginning of in “Linkage groups and syntenic regions of the genome” section. When null and non-synonymous mutants for a given gene are first introduced in a publication, the position of the original and replacement amino acids should be written in parentheses after the mutant haplotype identifier, e.g. *vrn-A1_m1* (missense, V6M) or *vrn-A1_m2* (null, W91*). For mutants generated using gene-editing technology (e.g. CRISPR-Cas9, TALENs), the bases that are altered or deleted should be described for each line, and if editing is used to alter a specific amino acid in the encoded protein, then this information should be provided. Given that alleles are based on plant or molecular phenotype, and as detailed in “Alleles” section, mutant haplotypes should only be assigned as novel (e.g. *_m1*, *m2*) if the mutation causes a unique change in the sequence of the locus that has not already been published for another allele.

In situations where an induced mutant is more widely important or used subsequently in breeding, then an allele designation could be warranted by the WGC with the *_mX* designation replaced by a lowercase letter, as described in “Alleles” section.

## Gene complexes

Gene complexes consist of functionally related genes that are genetically closely linked. Whether composed of a few or many genes, a gene complex should be assigned one symbol, in accordance with the procedures described in Guidelines for nomenclature of biochemical molecular loci in wheat and related species" section. The individual genes that compose gene complexes may be designated by adding a hyphen (−) and an Arabic numeral to the locus designation. For example, *GLU-A1-1* and *GLU-B1-1* designate, respectively, the A- and B-genome genes that encode the x-type glutenin-1 proteins, while *GLU-A1-2* and *GLU-B1-2* designate, respectively, the A- and B-genome genes that encode the y-type glutenin-1 proteins. Different alleles of genes that are components of gene complexes may be designated following the system described in “Genes specifying the structure of similar macromolecules” section but with the lowercase italic letter following the gene designation rather than the locus designation. For example, *Glu-A1-1a* designates the Chinese Spring A genome allele that encodes the x-type glutenin-1 protein.

Until recently, Triticeae enzyme and protein encoding genes were commonly initially identified and assigned designations based on studies of aneuploid strains that lack and/or contain extra copies of whole chromosomes or telosomes. Consequently, evidence could be obtained for production of two or more similar enzyme or protein promoters by one chromosome arm without genetic evidence as to whether the promoters are products of a single gene, of different genes that are members of a gene complex, or of two or more genes that are not members of a gene complex. In these situations, only one locus designation for similar proteins or enzymes was assigned to a chromosome arm until recombination evidence indicated otherwise. With the new genomic resources in Chinese Spring and multiple chromosome-scale assemblies of additional wheat cultivars or lines, researchers should include this information in their definition of gene complexes. Authors are expected to use these genomic tools and should also include the criterion used to define and name gene complexes in relevant publications.

## Pseudogenes

The term pseudogene refers to a genomic sequence that resembles another gene and is defective (i.e. the open reading frame includes a premature stop codon, is truncated, or highly degenerated compared to the functional allele(s)) (Vanin 1985; Cheetham et al. 2020). Pseudogenes may occur singly or as a cluster close to a functional copy of the gene (or elsewhere in the genome). Pseudogenes will take the form *_pX* following the relevant locus or allele designation; lowercase italicised *p* meaning pseudogene, and *X* being a sequential numeral. For example, *RG-B1_p1* might refer to the first documented pseudogene at the *RG1* (*RG-B1*) locus for glume colour on chromosome 1B. It is important to note that pseudogenes may be transcribed, and researchers should be aware of this when naming a pseudogene. Pseudogenes should be distinguished from alleles that involve copy number variation of genes, which should be named according to the guidelines detailed in “Alleles” section. A pseudogene should only be designated if it can be identified as such.

## Proteins

The basic symbol for a macromolecule should be identical to the basic symbol for the locus or loci that encode the macromolecule (see in “Gene name” section) except that each letter in the symbol should be a capital Roman letter. For a macromolecule encoded by the members of a homeologous set of loci, the phenotype symbol should consist of the basic symbol followed by a hyphen and the same Arabic numeral as in the genotype symbol, e.g. the products of the *ADH-1* homeologous set of gene loci are designated ADH-1. The protein homologues of this family should be named ADH-A1, ADH-B1 and ADH-D1, with no italics. For products where there is a locus and gene name in use (e.g. *VRN-A1* and *AP1-A1*), either can be used in non-italics for the protein name (e.g. VRN-A1, AP1-A1, see Table 1), with one version used consistently within a given publication.

## Symbols for DNA markers

This section describes nomenclature for genetic markers that are detected at the DNA level. The most common polymorphisms include insertion/deletion (indel) events and single nucleotide polymorphisms (SNPs) detected with PCR-based assays (e.g. KASP markers), genome-wide arrays (e.g. SNP chips) or direct sequencing (e.g. ddRAD, or skimGBS). The guidelines also include a historical section relating to DNA markers detected by hybridisation with DNA probes [e.g. RFLPs (restriction-fragment-length polymorphisms)] and by amplification with primers [e.g. RAPDs (random-amplified-polymorphic DNAs)] and STSs (sequence-tagged sites, including loci detected with sequenced RFLP clones, sequenced RAPDs and clones containing micro- and mini-satellites), and simple sequence repeats (SSRs).

### Basic symbol

Given the huge numbers and the multiple types of markers and sources that have emerged over the past few years some general guidelines for nomenclature of markers within publications are recommended. Regardless of exact name there are some common features that should be made available to all researchers including:

i. Detection platform used (e.g. KASP, Illumina array, Axiom array, skimGBS),
ii. Primer sequences (e.g. KASP) or the 100 bp surrounding the SNP position (e.g. Illumina array, Axiom array, skimGBS),
iii. Coordinates of the polymorphism which would currently be based on the Chinese Spring reference genome assembly and gene models. As new assemblies are published it will be important that the accession of the cultivar coordinate system (e.g. GCA_900519105.1) is clearly indicated in publication.
iv. Where possible, marker information should be deposited in a publicly accessible database (e.g. GrainGenes, EnsemblPlants, CerealsDB).

#### SNPs and derived markers for known protein-coding genes

Where possible, the position relative to the ATG start codon and excluding introns should be included in the marker name (when downstream of the start codon), alongside the nucleotide polymorphism. For example, a C/T polymorphism at position 2504 bp in the *GW2-B1* gene could be GW2-B1_C2504T. If there are multiple transcript isoforms for the gene, the isoform being used as a reference should be defined so that the position of polymorphism within a coding sequence can be determined. In the case of small deletions, these could be indicated with the start and end of the deleted sequence and the “del” designation (e.g. a 20-nucleotide deletion in *GW2-B1* from position 53 to 72 bp could be designated GW2-B1_53_72del. If the focus is on an amino acid substitution (e.g. a serine (S) to phenylalanine (F) substitution at position 1152 of the protein) a SNP can be named S1152F with the original amino acid (S) written first and the novel amino acid (F) written second, and a derived KASP marker could be Kasp_Sr13a_S1152F. Premature stop codons should be indicated by an asterisk (*; e.g. GW2-B1_C2510*). In cases where amino acid names could be confused with nucleotides (cysteine and cytosine; threonine and thymine, glycine and guanine, alanine and adenine), the three-letter code for amino acids should be used.

#### SNPs and derived markers—anonymous DNA sequences

Historically, these are named based on a laboratory or project code followed by a number maintained by that laboratory, e.g. sunKasp_85 for the 85^th^ marker maintained by the Bariana laboratory at Plant Breeding Institute, University of Sydney; or BA00334300 is a Bristol Axiom Array marker available on the Axiom® Wheat Breeder's Genotyping Array (Allen et al. 2017). Moving forward, we propose that the nomenclature of SNPs should use the following guidelines:

**Table:** 

Position in name	Description
1 to 3	Designate the reference assembly that the SNP is on. CHS stands for Chinese spring. Most pangenomes assemblies already have three-letter codes (e.g. JAG for Jagger)
4 to 5	Sub-version of the assembly (e.g. version 2.1 of Chinese Spring would be designated 21 across characters 4 and 5)
6	Understroke
7 to 8	Chromosome and sub-genome
9 to 17	Position on the assembly with leading zeros used to standardise the format of positions

For example, CHS21_6A001234567 would be a SNP based on the Chinese Spring (CHI) version 2.1 assembly, chromosome 6A, base-pair position 1,234,567.

### Historical marker symbols

Historical marker symbols are provided here as Supplementary File 1 only as a reference and a guide to understand older studies. Going forward, their usage is discouraged.

## Symbols for loci and alleles controlling quantitative characters

### Genes identified by segregation analysis

Symbols for loci and alleles controlling quantitative characters that are identified by segregation analysis should be in accord with the Recommended Rules for Gene Symbolisation in Wheat.

### Quantitative trait loci (QTL)

QTL are loci controlling quantitative characters whose allelic classes do not exhibit discontinuous variation or clear segregation patterns. They are identified by association with one or more linked markers.

#### Basic symbol

The basic symbol for QTL should be “Q”.

#### Locus symbols

The “Q” should be followed by a trait designator, a period, a laboratory designator (see in "SNPs and derived markers—anonymous DNA sequences" section), a hyphen (−) and the symbol for the chromosome in which the QTL is located. The trait designator should consist of no more than four and preferably three letters, the first of which is uppercase.

Different QTLs for the same trait that are identified in one chromosome should be assigned the same symbol except for the addition of a period and an Arabic numeral after the chromosome designation. All characters in the locus symbol should be italicised. For example, *QYld.psr-7B.1* and *QYld.psr-7B.2* would designate two yield QTL identified in chromosome 7B by the John Innes Centre. On a map of 7B, these could be abbreviated as *QYld.psr.1* and *QYld.psr.2.*

#### Allele symbols

Alleles at QTL loci should be designated by a lowercase italic letter following the locus designation.

## Guidelines for nomenclature of genes for reaction to pathogenic diseases and pests

### Locus designation

All loci will have uppercase letters; all alleles conferring resistance (low reaction) will be designated with an uppercase first letter, even though some might inherit as recessive alleles. Moreover, the dominance of individual alleles may vary with the environment, the genetic background and the particular culture of the pathogen. Symbols for disease/pest-reaction genes are used by people of many disciplines, and since they are frequently communicated verbally, dominance relationships are not clear. Those resistance alleles initially designated with a lowercase first letter have tended to be miswritten with an uppercase first letter. For example, for *Sr17* the usually recessive resistance allele *Sr17* was initially designated *sr17* but its presentation in some reports was confusing.

### Loci conferring multiple disease/pest reactions

Where no recombination occurs between genes conferring resistance to more than one disease response locus, the gene(s) segment shall be designated separately for each one, e.g. *PM1, SR15* and *LR20*. Mutation and cloning showed that *PM1* is a different locus (Hewitt et al. 2021).

### Reaction loci defined by recombination

Where recombination occurs between two closely linked factors for reaction to a pathogen, the recombined “allele” may be designated as a combination of the differently designated alleles, e.g. the recombined “allele” obtained by combining *Lr14a* and *Lr14b* was designated as *Lr14ab*. The decision as to whether a designation should be as a combination or as separate genes shall be at the discretion of the investigators. A maximum value of 1 crossover unit for designation as an “allele” is suggested.

### Naming corresponding genes in pathogens/pests

Although the need to consider uniform symbolisation of corresponding genes in pathogens and pests is recognised, no recommendations are proposed. From a wheat perspective, *AvrLr14a* might be acceptable, but specialists working with *Puccinia triticina* as a different organism might have other opinions.

## Guidelines for nomenclature of genes underlying variation in proteins and enzymes

The majority of characterised proteins and enzymes in wheat are encoded by gene sets homologous to genes studied in other species, and are therefore named according to "Genes with similar phenotypic effects" section. The alleles of these genes are frequently defined by molecular phenotype (In “Alleles” section) identified using a range of complementary techniques (including 1D and 2D protein electrophoresis, mass spectrometry, chromatography, DNA markers and/or DNA sequencing), given that in many cases it is difficult to distinguish alleles using a single method; for example (Liu et al. 2010; Igrejas et al. 2020), different combinations of four methods (SDS-PAGE, IEF x SDS-PAGE, MALDI-TOF-MS and PCR) were required to distinguish alleles across the three *GLU-3* loci. For a gene category where there is a predominant phenotyping method, this is included in the introductory paragraphs to the sub-section and notes added only to alleles where alternative methods of identification were employed. Genetic differences for functional quality properties are generally complex and are listed under traits in the Morphological and Physiological section of the Catalogue, although some of the underlying genes, such as those for flour colour, are listed in the Protein section.

In general, non-functional DNA variants (synonymous variations in coding regions) will be designated as haplotypes, as elsewhere in the Catalogue (*_h1*, *_h2*, etc.). In contrast, where, for example, protein electrophoresis fails to identify differences in mobility between bands shown to be different by DNA sequencing, such variants could be directly included as alleles (non-synonymous variations). Application of an alternative definition of haplotype will be explained by notes; for example, defining a haplotype as a combination of alleles at more than one locus, as in the case of (i) glutenin subunit combinations encoded by alleles of the *GLU-1–1* and *GLU-1–2* loci, and (ii) allelic combinations of the *GLU-3* and *GLI-1* loci.

Gene model identifiers for individual genes will be incorporated into the catalogue. Conversely, the locus/gene designations of the catalogue will be incorporated into the available sequenced reference genomes, along with the name of the gene product (e.g. high-molecular-weight glutenin, ω-gliadin, alcohol dehydrogenase). Cross-referencing to the genetic nomenclatures of other members of the Triticeae and beyond is encouraged.

A link of a named gene to a germplasm stock in an international and publicly accessible GenBank as outlined in "Germplasm" section is strongly encouraged.

## Germplasm

Wheat is an important food and industrial crop as well as a model allopolyploid organism. Germplasm is a key component of the Gene Catalogue, and it is expected that the germplasm associated with any formally named gene is clearly defined and referenced to an internationally accessible germplasm collection. This collection *must* allow worldwide access to the germplasm within the framework of the Food and Agriculture Organization of the United Nations International Treaty on Plant Genetic Resources for Food and Agriculture. Specialists in the rusts, powdery mildew and some other wheat diseases have adopted a procedure of pre-publication approval of gene names and assurance of germplasm availability as a basis for a permanent gene name. When specific reference germplasm cannot be assured, proposers are encouraged to use temporary names.

## Discussion

These guidelines provide an updated framework for consistent naming of genes and genomic regions in wheat. These guidelines are flexible and not overly prescriptive or static, providing a common reference point for the wheat community. The revised guidelines aim to accommodate historical nomenclature conventions while also adapting to new advances in genomics and biotechnology. With new genome assemblies and gene annotations, we urge researchers to adopt these common standards and to provide the necessary information on the naming of genes, alleles, haplotypes, markers, and QTL when reporting their findings. We particularly encourage colleagues who are new to wheat research to adopt these guidelines when naming genes or alleles, rather than following conventions used in other species. As new technology and biological understanding are generated, there will undoubtedly be further reviews and changes necessary when shortcomings emerge and new approaches are required. These guidelines should facilitate integration of data from independent studies, allow more efficient text and data mining approaches, and ultimately accelerate wheat research. The Wheat Gene Catalogue is currently hosted by KOMUGI (https://shigen.nig.ac.jp/wheat/komugi/genes/symbolClassList.jsp ) and at GrainGenes (https://wheat.pw.usda.gov/ggpages/awn/; Blake et al. 2019), the latter also providing ongoing curation. In time, these guidelines may also be adopted by the barley, rye, triticale, and oat research communities, for which similar advances are being made in deciphering their genome sequences; a common set of guidelines would help transfer knowledge between these cereals, which share close phylogenetic relationships and share common breeding targets. We hope these guidelines provide an informative reference for researchers during this exciting era of wheat science, in which we believe important advances will continue to be made in our understanding of genes that control agronomically important traits.

## Supplementary Information

Below is the link to the electronic supplementary material.

Supplementary file1 (PDF 125 kb)

## Data Availability

The authors declare that there is no data associated with these guidelines.

## References

[CR1] Abrouk M, Athiyannan N, Müller T, Pailles Y, Stritt C, Roulin AC, Chu C, Liu S, Morita T, Handa H, Poland J, Keller B, Krattinger SG (2021). Population genomics and haplotype analysis in spelt and bread wheat identifies a gene regulating glume color. Commun Biol.

[CR2] Allen AM, Winfield MO, Burridge AJ (2017). Characterization of a wheat Breeders’ Array suitable for high-throughput SNP genotyping of global accessions of hexaploid bread wheat (*Triticum aestivum*). Plant Biotech J.

[CR3] Anonymous 1979 Enzyme Nomenclature (1978) Recommendations of the Nomenclature Committee of the International Union of Biochemistry. Academic Press, New York

[CR4] Anonymous 1986 Enzyme Nomenclature (1986) Recommendations of the Nomenclature Committee of the International Union of Biochemistry. Academic Press, New York

[CR5] Beales J, Turner A, Griffiths S, Snape JW, Laurie DA (2007). A pseudo-response regulator is mis-expressed in the photoperiod insensitive *Ppd-D1a* mutant of wheat (*Triticum aestivum* L.). Theor Appl Genet.

[CR6] Blake VC, Woodhouse MR, Lazo GR, Odell SG, Wight CP, Tinker NA, Wang Y, Gu YQ, Birkett CL, Jannink JL, Matthews DE, Hane DL, Michel SL, Yao E, Sen TZ (2019). GrainGenes: centralized small grain resources and digital platform for geneticists and breeders. Database.

[CR7] Cheetham SW, Faulkner GJ, Dinger ME (2020). Overcoming challenges and dogmas to understand the functions of pseudogenes. Nat Rev Genet.

[CR8] Debernardi JM, Lin H, Chuck G, Faris JD, Dubcovsky J (2017). *microRNA172* plays a crucial role in wheat spike morphogenesis and grain threshability. Development.

[CR9] Debernardi JM, Tricoli DM, Ercoli MF, Hayta S, Ronald P, Palatnik JF, Dubcovsky J (2020). A GRF-GIF chimeric protein improves the regeneration efficiency of transgenic plants. Nat Biotechnol.

[CR10] Driscoll CJ, Sears ER (1965). Mapping of a wheat-rye translocation. Genetics.

[CR11] Dvorak J, Deal KR, Luo MC (2006). Discovery and mapping of wheat *Ph1* suppressors. Genetics.

[CR12] Gale M, Devos K (1998). Comparative genetics in the grasses. Proc Natl Acad Sci U S A.

[CR13] Gaurav K, Arora S, Silva P, Sanchez-Martin J, Horsnell R, Gao L, Brar GS, Widrig V, John Raupp W, Singh N, Wu S, Kale SM, Chinoy C, Nicholson P, Quiroz-Chavez J, Simmonds J, Hayta S, Smedley MA, Harwood W, Pearce S, Gilbert D, Kangara N, Gardener C, Forner-Martinez M, Liu J, Yu G, Boden SA, Pascucci A, Ghosh S, Hafeez AN, O’Hara T, Waites J, Cheema J, Steuernagel B, Patpour M, Justesen AF, Liu S, Rudd JC, Avni R, Sharon A, Steiner B, Kirana RP, Buerstmayr H, Mehrabi AA, Nasyrova FY, Chayut N, Matny O, Steffenson BJ, Sandhu N, Chhuneja P, Lagudah E, Elkot AF, Tyrrell S, Bian X, Davey RP, Simonsen M, Schauser L, Tiwari VK, Randy Kutcher H, Hucl P, Li A, Liu DC, Mao L, Xu S, Brown-Guedira G, Faris J, Dvorak J, Luo MC, Krasileva K, Lux T, Artmeier S, Mayer KFX, Uauy C, Mascher M, Bentley AR, Keller B, Poland J, Wulff BBH (2022). Population genomic analysis of *Aegilops tauschii* identifies targets for bread wheat improvement. Nat Biotechnol.

[CR14] Hart GE (1987) Genetic and biochemical studies of enzymes. In: Heyne EG (ed.) Wheat and Wheat Improvement. pp 199–214. American Society of Agronomy, Madison

[CR15] Hewitt T, Muller MC, Molnar I, Mascher M, Holusova K, Simkova H, Kunz L, Zhang JP, Li JB, Bhatt D, Sharma R, Schudel S, Yu GT, Steurnagel B, Periyannan S, Wulff B, Ayliffe M, McIntosh R, Keller B, Lagudah E, Zhang P (2021). A highly differentiated region of wheat chromosome 7AL encodes a *Pm1a* immune receptor that recognizes its corresponding *AvrPm1a* effector from *Blumeria graminis*. New Phytol.

[CR16] Igrejas G, Ikeda TM, Guzman C (2020). Wheat quality for improving processing and human health.

[CR17] International Wheat Genome Sequencing Consortium (2018). Shifting the limits in wheat research and breeding using a fully annotated reference genome. Science.

[CR18] Ishida Y, Tsunashima M, Hiei Y, Komari T, Wang K (2015). Wheat transformation. Agrobacterium protocols: methods in molecular biology.

[CR19] Kihara H (1944). Discovery of the DD analyser, one of the ancestors of *Triticum vulgare* (in Japanese). Agri Horti.

[CR20] Koebner RMD, Miller TE (1986). A note on the nomenclature for translocated chromosomes in the Triticeae. Cereal Res Commun.

[CR21] Krasileva KV, Vasquez-Gross HA, Howell T, Bailey P, Paraiso F, Clissold L, Simmonds J, Ramirez-Gonzalez RH, Wang X, Borrill P, Fosker C, Ayling S, Phillips AL, Uauy C, Dubcovsky J (2017). Uncovering hidden variation in polyploid wheat. Proc Natl Acad Sci USA.

[CR22] Ling HQ, Ma B, Shi X, Liu H, Dong L, Sun H, Cao Y, Gao Q, Zheng S, Li Y, Yu Y, Du H, Qi M, Li Y, Lu H, Yu H, Cui Y, Wang N, Chen C, Wu H, Zhao Y, Zhang J, Li Y, Zhou W, Zhang B, Hu W, van Eijk MJT, Tang J, Witsenboer HMA, Zhao S, Li Z, Zhang A, Wang D, Liang C (2018). Genome sequence of the progenitor of wheat A subgenome *Triticum urartu*. Nature.

[CR23] Liu L, Ikeda TM, Branlard G, Peña RJ, Rogers WR, Lerner SE, Kolman MA, Xia XC, Wan LH, Ma WJ, Appels R, Yoshida H, Wang AL, Yan YM, He ZH (2010). Comparison of low molecular weight glutenin subunits identified by SDS-PAGE, 2-DE, MALDI-TOF-MS and PCR in common wheat. BMC Plant Biol.

[CR24] Maccaferri M, Harris NS, Twardziok SO, Pasam RK, Gundlach H, Spannagl M, Ormanbekova D, Lux T, Prade VM, Milner SG, Himmelbach A, Mascher M, Bagnaresi P, Faccioli P, Cozzi P, Lauria M, Lazzari B, Stella A, Manconi A, Gnocchi M, Moscatelli M, Avni R, Deek J, Biyiklioglu S, Frascaroli E, Corneti S, Salvi S, Sonnante G, Desiderio F, Marè C, Crosatti C, Mica E, Özkan H, Kilian B, De Vita P, Marone D, Joukhadar R, Mazzucotelli E, Nigro D, Gadaleta A, Chao S, Faris JD, Melo ATO, Pumphrey M, Pecchioni N, Milanesi L, Wiebe K, Ens J, MacLachlan RP, Clarke JM, Sharpe AG, Koh CS, Liang KYH, Taylor GJ, Knox R, Budak H, Mastrangelo AM, Xu SS, Stein N, Hale I, Distelfeld A, Hayden MJ, Tuberosa R, Walkowiak S, Mayer KFX, Ceriotti A, Pozniak CJ, Cattivelli L (2019). Durum wheat genome highlights past domestication signatures and future improvement targets. Nat Genetics.

[CR25] McCouch SR, Committee on Gene Symbolization, Nomenclature and Linkage, Rice Genetics Cooperative (2008) Gene nomenclature system for rice. Rice 1:72–84

[CR26] McIntosh R, Yamazaki Y, Dubcovsky J, Rogers J, Morris C, Appels R, Xia XC (2013) Catalogue of gene symbols for wheat. In: 12th International wheat genetics symposium 8–13 September 2013 Yokohama, Japan

[CR27] Moore G, Devos K, Wang Z, Gale M (1995). Grasses, line up and form a circle. Curr Biol.

[CR28] Morris R, Sears ER (1967) The cytogenetics of wheat and its relatives. In: Quisenberry KS & Reitz LP (eds) Wheat and wheat improvement. pp19–87. American Society of Agronomy, Madison

[CR29] Peng J, Richards DE, Hartley NM, Murphy GP, Devos KM, Flintham JE, Beales J, Fish LJ, Worland AJ, Pelica F, Sudhakar D, Christou P, Snape JW, Gale MD, Harberd NP (1999). ‘Green revolution’ genes encode mutant gibberellin response modulators. Nature.

[CR30] Ramirez-Gonzalez RH, Borrill P, Lang D, Harrington SA, Brinton J, Venturini L, Davey M, Jacobs J, van Ex F, Pasha A, Khedikar Y, Robinson SJ, Cory AT, Florio T, Concia L, Juery C, Schoonbeek H, Steuernagel B, Xiang D, Ridout CJ, Chalhoub B, Mayer KFX, Benhamed M, Latrasse D, Bendahmane A, International Wheat Genome Sequencing C, Wulff BBH, Appels R, Tiwari V, Datla R, Choulet F, Pozniak CJ, Provart NJ, Sharpe AG, Paux E, Spannagl M, Brautigam A, Uauy C (2018) The transcriptional landscape of polyploid wheat. Science 361:eaar6089 10.1126/science.aar6089 30115782

[CR31] Schilling S, Kennedy A, Pan S, Jermiin LS, Melzer R (2020). Genome-wide analysis of MIKC-type MADS-box genes in wheat: pervasive duplications, functional conservation and putative neofunctionalization. New Phytol.

[CR32] Sears ER (1948). The Cytology and genetics of the wheats and their relatives. Advanced Genetics.

[CR33] Sears ER (1954). The aneuploids of common wheat. Missouri Agric Exp Stn Res Bull.

[CR34] Sears ER (1967). Induced transfer of hairy neck from rye to wheat. Zeitschrift Fur Pflanzenzuchtung.

[CR35] Shiferaw B, Smale M, Braun H-J, Duveiller E, Reynolds M, Muricho G (2013). Crops that feed the world 10. Past successes and future challenges to the role played by wheat in global food security. Food Secur.

[CR36] Simons KJ, Fellers JP, Trick HN, Zhang Z, Tai YS, Gill BS, Faris JD (2006). Molecular characterization of the major wheat domestication gene *Q*. Genetics.

[CR37] Tsunewaki K, Ogihara Y, Takumi S, Handa H (2015). Prof. H. Kihara's genome concept and advancements in wheat cytogenetics in his school. Advances in wheat genetics: from genome to field.

[CR38] Vanin EF (1985). Processed pseudogenes: characteristics and evolution. Ann Rev Genet.

[CR39] Walkowiak S, Gao L, Monat C, Haberer G, Kassa MT, Brinton J, Ramirez-Gonzalez RH, Kolodziej MC, Delorean E, Thambugala D, Klymiuk V, Byrns B, Gundlach H, Bandi V, Siri JN, Nilsen K, Aquino C, Himmelbach A, Copetti D, Ban T, Venturini L, Bevan M, Clavijo B, Koo DH, Ens J, Wiebe K, N'Diaye A, Fritz AK, Gutwin C, Fiebig A, Fosker C, Fu BX, Accinelli GG, Gardner KA, Fradgley N, Gutierrez-Gonzalez J, Halstead-Nussloch G, Hatakeyama M, Koh CS, Deek J, Costamagna AC, Fobert P, Heavens D, Kanamori H, Kawaura K, Kobayashi F, Krasileva K, Kuo T, McKenzie N, Murata K, Nabeka Y, Paape T, Padmarasu S, Percival-Alwyn L, Kagale S, Scholz U, Sese J, Juliana P, Singh R, Shimizu-Inatsugi R, Swarbreck D, Cockram J, Budak H, Tameshige T, Tanaka T, Tsuji H, Wright J, Wu J, Steuernagel B, Small I, Cloutier S, Keeble-Gagnere G, Muehlbauer G, Tibbets J, Nasuda S, Melonek J, Hucl PJ, Sharpe AG, Clark M, Legg E, Bharti A, Langridge P, Hall A, Uauy C, Mascher M, Krattinger SG, Handa H, Shimizu KK, Distelfeld A, Chalmers K, Keller B, Mayer KFX, Poland J, Stein N, McCartney CA, Spannagl M, Wicker T, Pozniak CJ (2020). Multiple wheat genomes reveal global variation in modern breeding. Nature.

[CR40] Werner JR, Endo TR, Gill BS (1992). Toward a cytogenetically based physical map of the wheat genome. Proc Natl Acad Sci USA.

[CR41] Woodhouse MR, Cannon EK, Portwood JL, Harper LC, Gardiner JM, Schaeffer ML, Andorf CM (2021). A pan-genomic approach to genome databases using maize as a model system. BMC Plant Biol.

[CR42] Yan L, Loukoianov A, Tranquilli G, Helguera M, Fahima T, Dubcovsky J (2003). Positional cloning of the wheat vernalization gene *VRN1*. Proc Natl Acad Sci USA.

