# Supplementary material for: Updated guidelines for gene nomenclature in wheat
Source: Theor Appl Genet. 2023 Mar 23;136(4):72. doi: 10.1007/s00122-023-04253-w (PMC10036449; doi:10.1007/s00122-023-04253-w)
Supplement: Supplementary file 1 — Supplementary file1 (PDF 125 kb) [file 122_2023_4253_MOESM1_ESM.pdf]

Updated guidelines for gene nomenclature in wheat

Supplementary file 1: Historical marker symbols

6.2 Historical marker symbols: Historical marker symbols are provided only as a reference and a guide to understand older studies. Going forward, their usage is discouraged.

6.2.1 *Locus symbols:* The basic symbol for the genetic map location of DNA markers with unknown function should be prefixed with an 'X'. The 'X' should be followed by a laboratory designator (see Section 6.1.2 and 7), a number that identifies the probe or primer(s) used to detect the locus, a hyphen (-), and the symbol for the chromosome in which the locus is located. The laboratory designator and number should be assigned by the laboratory that produced the clone or sequenced the primer(s) or, if that laboratory chooses not to do so, then by the laboratory that mapped the locus. The number should consist of one or more Arabic numerals and should begin with a numeral other than zero, i.e., numbers such as '01', '001', and '002' should not be used. The number assigned to a probe need bear no relationship to the name of the clone used to produce the probe and, likewise, the number assigned to a primer(s) need bear no relationship to the name of the clone used to produce the probe and, likewise, the number assigned to a primer(s) need bear no relationship to any name that may have been assigned to the primer(s). The letters in the laboratory designator should be lower-case and all characters in the locus symbol should be italicized. For example, *Xpsr119-7A* designates an RFLP locus located on chromosome 7A detected with *P*lant *S*cience *R*esearch probe 119 of the John Innes Centre. DNA markers detected in different chromosomes with the same probe or primer(s) should be assigned the same symbol except for the chromosome designation. For example, *Xpsr119-7D* and *Xpsr119-4A* designate other loci detected with probe PSR119; *Xwmc240-2A* is the first locus on chromosome 2A detected with primer pair WMC240.

6.2.2 *Locus symbols for DNA markers detected with 'known function' probes or with primers that amplify genes:* The locus symbols for RFLP markers of unknown function that are detected with 'known-function' probes may include, in parentheses following the probe number, a symbol for the gene from which the probe was obtained. For example, *Xprsr804(SBP)-3A* designates a chromosome 3A locus detected with a sedoheptulose-1,7-bisphosphase gene probe. Likewise, when the primers used to amplify a DNA marker of unknown-function are of sufficient length and similarity to a known gene to amplify the gene, the DNA-marker symbol may include the gene symbol in parentheses following the number assigned to the primers. For genes where the Commission on Plant Gene Nomenclature has assigned mnemonic designations, the set number and other numbers assigned by the Commission may also be included inside the parentheses immediately after the gene symbol.

6.3 'Known-function' DNA markers: Loci that are detected with a DNA probe or DNA primers and whose function has been demonstrated should be designated with a symbol that indicates the function of the locus, as described in either Section 2 or in the Recommended Rules for Gene Symbolization in Wheat. It must be emphasized, however,

that some clones and primers are likely to detect both loci whose function is known (proven, for example, by a segregation test against allelic forms of a gene encoding a protein) and additional loci of unknown (i.e., unproven) function (either pseudogenes or unrelated loci whose sequence homology to the probe or primers is sufficient to allow detection by it). In this case, the two types of loci require different nomenclature, namely, that described in Section 2 or in the Recommended Rules for Gene Symbolization in Wheat (https://wheat.pw.usda.gov/ggpages/wgc/98/Intro.htm) and in Section 4, respectively.

6.4 Duplicate DNA-marker loci: DNA markers located in the same chromosome that hybridize with the same probe or that are amplified with the same primer(s) should be assigned the same symbol except for the addition of a period and an Arabic numeral immediately after the chromosome designation. For example, *Xpsr933-2A.1* and *Xpsr933-2A.2* designate duplicate loci located on chromosome 2A that are detected with probe PSR933. As when two or more enzyme or protein promoters are produced by one chromosome arm, multiple DNA fragments from one chromosome arm that hybridize to one probe or that are amplified by one pair of primers (or by one primer) should be assigned to only one locus until recombination evidence indicates otherwise. As noted in Section 6, DNA markers located in different chromosomes that hybridize with the same probe or that are amplified with the same primer(s) should be assigned the same symbol except for the chromosome designation.

6.5 Allele symbols: Alleles should be designated as outlined in Section 2.5 with the exception that restriction-enzyme-specific alleles, e.g., RFLP- and indirect-STS alleles, should be designated with the name of the restriction enzyme followed by a lower-case letter. For example, *Xtam-5A_HindIIIa* denotes an allele detected with *Hind*III. Where possible, Chinese Spring should be the prototype for allele '*a*'. When a double-digest is used to detect an allele, both restriction enzymes should be listed, separated by a slash. The name and source of the probe or primer(s) and the length(s) of the DNA fragment(s) detected normally should be stated in the first publication describing an allele.

6.6 Abbreviation of locus and allele symbols: The chromosome designation is an integral part of the locus symbol for DNA markers. Nevertheless, on chromosome maps and in a limited number of other contexts, the chromosome designation and the hyphen preceding it may be omitted. For example, *Xpsr34-3A* may be abbreviated as *Xpsr34* on a map of chromosome 3A, *Xpsr933-2A.1* and *Xpsr933-2A.2,* respectively, may be abbreviated as *Xpsr933.1* and *Xpsr933.2* on a map of 2A, and *Xpsr804(SHP)-3A* may be abbreviated as *Xpsr804(SHP)* on a map of 3A. Finally, *Xbgl485(GER)-4D.2* may be abbreviated on a map of 4D by omission of the hyphen, the chromosome designation, and the period, i.e., as *Xbgl485(GER).2.* In some situations, it will also be possible to abbreviate the symbols for alleles as, for example, *BamH1b*, or even simply *b*.

6.7 Laboratory designators: Laboratory designators should consist of two to four (preferably three) letters. When used in locus symbols, all letters should be lowercase and italicized (see Section 6.1.2). Lists of laboratory designators are no longer maintained. Laboratory designators should be chosen carefully to ensure that they differ both from those used by other laboratories and from those that compose gene symbols. As an aid in this regard, a

list of laboratory designators that have appeared in the literature is available electronically at https://wheat.pw.usda.gov/ggpages/Lab.Designators.html.Laboratories that are investigating DNA markers in different species and/or different types, e.g., RFLPs, STS, RAPDs, SSR, KASP may choose to use more than one designator. For example, oat and barley cDNA clones isolated at Cornell University have been designated with the prefixes CDO and BCD, respectively, and *cdo* and *bcd*, respectively, are appropriately used as laboratory designators in symbols for loci detected with these clones. Likewise, *tam* and *txs*, respectively, are being used as laboratory designators in symbols for loci detected with wheat and sorghum DNA clones isolated at Texas A&M University, and the John Innes Centre is using *psr* and *psm* as laboratory designators in the symbols for DNA markers detected with wheat and millet probes, respectively, and *psp* for wheat PCR markers.

6.8 Clone designations: Clone designations should minimally identify the type of vector, the species from which the cloned DNA was obtained, and the source laboratory and cloned DNA, in that order. P = plasmid, l = lambda, c = cosmid, and m = M13 should be used to identify vectors. Initials of the species name, e.g., TA = *Triticum aestivum* and SC = *Secale cereale*, should be used to designate the cloned DNA and a unique letter-number combination chosen by the source laboratory should be used to designate the source laboratory and cloned DNA.
